# Real-World Use of Albutrepenonacog Alfa, A Recombinant Coagulation Factor IX Albumin Fusion Protein, for Personalized Prophylaxis in Japanese Individuals With Hemophilia B: A Case Series

**DOI:** 10.7759/cureus.33573

**Published:** 2023-01-09

**Authors:** Azusa Nagao, Masato Bingo, Tomoko Yamaguchi, Katsuyuki Fukutake

**Affiliations:** 1 Department of Blood Coagulation, Ogikubo Hospital, Tokyo, JPN; 2 Department of Laboratory Medicine, Tokyo Medical University Hospital, Tokyo, JPN

**Keywords:** albutrepenonacog alfa, factor xi, hemophilia b, personalized usage, prophylaxis, real-world clinical practice

## Abstract

Currently, the mainstay of disease management for hemophilia B, a hemorrhagic disease caused by a congenital deficiency or molecular abnormalities of blood coagulation factor IX (FIX), is prophylaxis using FIX concentrate. On-demand injections of FIX concentrate may also be required, even during prophylaxis, when a patient with hemophilia B is bleeding. Albutrepenonacog alfa (rFIX-FP) is a human albumin fusion gene recombinant FIX, which is administered once every seven, 14, or 21 days, depending on patient preferences and symptoms. Studies have demonstrated its efficacy and safety in a range of patients; however, to date, reports of real-world experiences of the use of rFIX-FP in Japan are limited. We present a case series of three Japanese individuals with moderately severe (FIX activity 1 to <2%) or severe (FIX activity <1%) hemophilia B who benefited from prophylaxis using rFIX-FP in our clinical practice setting. We highlighted the good effectiveness of rFIX-FP in a patient with moderately severe hemophilia B who required prophylaxis due to joint bleeding, which was causing deterioration of his left ankle joint, as well as in a patient with severe hemophilia B and atherothrombotic cerebral infarction, whose trough level had to be ≥5% for concomitant use of an antiplatelet drug, and in a patient with severe hemophilia B who was working in nursing care, which involved heavy labor and night shifts, and who had previously been treated with on-demand FIX concentrate. In all three cases, rFIX-FP improved disease symptoms, and the patients were able to maintain steady states of therapy due to the treatment characteristics of rFIX-FP, which stabilizes FIX at high trough levels.

## Introduction

Hemophilia B is a hemorrhagic disease caused by a congenital deficiency of blood coagulation factor IX (FIX) or molecular abnormalities [1]. The mainstay of hemophilia B disease management, in particular, for those with severe hemophilia (defined as FIX activity of <1%) or with moderate hemophilia (FIX activity of 1% to <5%) with a severe phenotype, is prophylaxis with FIX, which is recommended in Japanese and international guidelines [1-3]. In fact, in Japan, the implementation rate of prophylaxis was as high as 87.0% for individuals with severe hemophilia B in 2020 [4].

FIX is administered intravenously and as frequent intravenous injections place a burden on patients, FIX therapies with prolonged half-lives have been developed [5]. One such product is albutrepenonacog alfa (Idelvion®, CSL Behring; hereafter called “rFIX-FP”), a human albumin fusion gene recombinant FIX, which is typically administered once every 7-14 days or 21 days, depending on bleeding symptoms [6]. The efficacy and safety of rFIX-FP, administered as part of a prophylaxis regimen or as an on-demand treatment, has been demonstrated in both a phase II/III study (3001 study) [7] and a phase III study (3002 study) [8]. Furthermore, the effectiveness of rFIX-FP in actual clinical practice has been demonstrated in the United States and Europe [9,10]. However, to date, there are limited real-world data on the use of rFIX-FP in Japan [11].

To address this knowledge gap, we describe the characteristics and treatment of three individuals with hemophilia B who received regular replacement therapy with rFIX-FP at the Ogikubo Hospital in Tokyo, Japan.

## Case presentation

Case 1: A patient with moderate Hemophilia B who required prophylactic treatment due to joint bleeding

**Figure 1:**
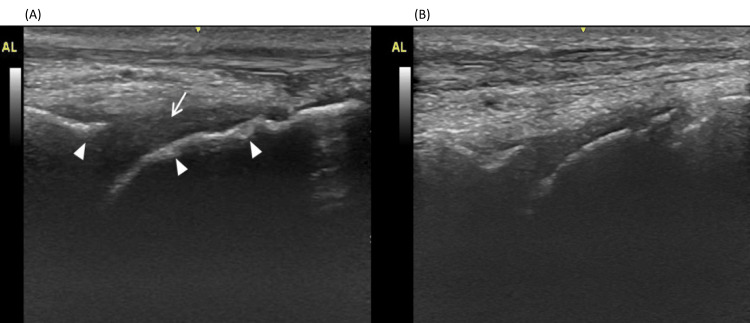
Ultrasonographic images of (A) Case 1 in September 2019 before albutrepenonacog alfa (rFIX-FP) treatment; image shows the left ankle joint, where liquid storage (arrow) and osteochondral damage (arrowheads) are observed and (B) in April 2020 while receiving rFIX-FP 3500 units (60.3 IU/kg) once weekly.

**Figure 2:**
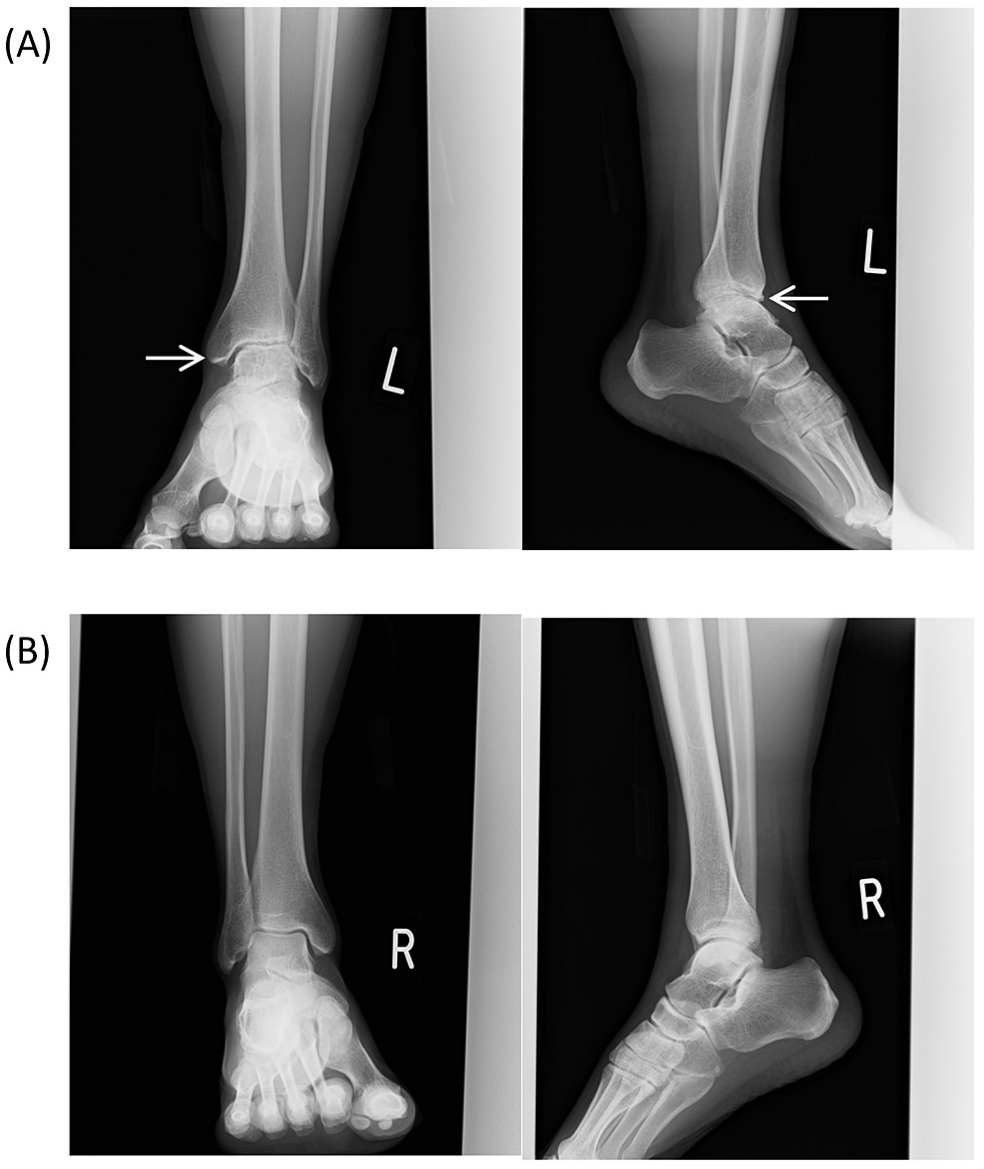
Joint X-ray images of Case 1 (A) left ankle joint showing advanced stage arthropathy (arrow) and (B) right ankle joint in September 2019 before albutrepenonacog alfa (rFIX-FP) treatment.

A male patient in his 30s with moderately severe hemophilia B (FIX activity 2%) and a body weight of 58 kilograms (kg) made an initial visit to our hospital in December 2017. At this time, non-articular bleeding occurred once or twice a month, and he was receiving on-demand therapy with plasma-derived human coagulation FIX, which was subsequently switched to nonacog alfa. Over the two years from 2017 to 2019, the patient experienced two hemorrhages in his left ankle; however, as these hemorrhages were considered mild, prophylactic treatment was not considered at the time. In September 2019, the patient presented with persistent discomfort in the left ankle joint for the previous month. Joint ultrasonography showed bleeding in the joint (Figure 1) and joint X-ray showed advanced-stage arthropathy (Figure 2). This was the first time an X-ray was performed on this patient, as his previous experience with joint hemorrhage was limited. However, considering that the patient seemed to have had repeated bleeding episodes in the past and still had active bleeding, prophylaxis was proposed. Given his age and the fact that this was his first use of prophylaxis, the patient was switched to treatment with rFIX-FP, as he did not want to receive twice-weekly replacement therapy with nonacog alfa. As such, rFIX-FP 2000 units (34.5 IU/kg) were administered once weekly from September 2019 until January 2020. During this period, five bleeding episodes in the left ankle joint were observed. Joint ultrasonography showed no improvement in his arthropathy due to fluid accumulation in the joint, so the dose was increased to 3500 units (60.3 IU/kg) once weekly from February 2020. In April 2020, improvement in joint bleeding was confirmed by ultrasonography, and therefore the dosing interval was extended to once every two weeks with 3500 units from May of the same year. As of August 2021, the patient continued to receive prophylaxis with the same regimen without new bleeding.

Case 2: A patient with severe Hemophilia B whose FIX trough level had to be ≥5% for concomitant use of an antiplatelet drug

A male patient in his 40s (body weight 69 kg) had severe hemophilia B (FIX activity <1%) complicated by HIV infection, hypertension, diabetes mellitus, hepatitis C with a sustained virologic response, and alcoholic liver disorder. He started prophylaxis with self-injection of rFIX-FP 4000 units (58.8 IU/kg) once every two weeks in December 2017, and almost no bleeding was observed. In December 2019, due to the patient developing atherothrombotic cerebral infarction, daily oral aspirin 100 mg was introduced to avoid the reoccurrence of infarction. When antiplatelet drugs are administered to patients with hemophilia, prophylaxis needs to be concomitantly performed with strictly controlled trough level of coagulation factors for the prevention of major bleeding complications [12]. As such, the dose of rFIX-FP was changed to 2000 units (29.4 IU/kg) once weekly so that the dose could be flexibly adjusted according to the clinical course based on the World Federation of Hemophilia guidelines [1], which recommend targeting a FIX trough level of 5% or higher. As of March 2020, the patient’s FIX trough level had reached 7%. There has been no significant bleeding, joint bleeding, or recurrence of thrombosis. The patient continued to experience hemiplegia, so once-weekly self-treatment with rFIX-FP is still underway and performed under the supervision of a visiting nurse.

Case 3: A patient who engaged in heavy labor and in whom prophylactic treatment had not been introduced

**Figure 3:**
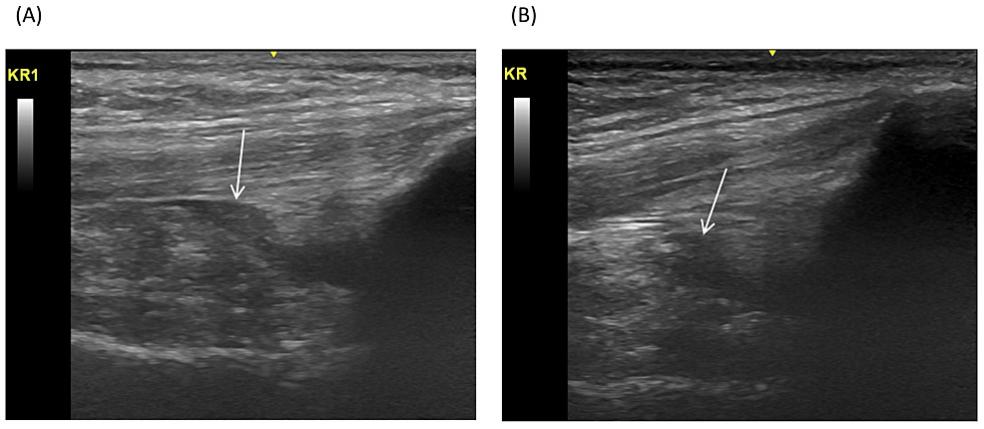
Ultrasonographic image of the right knee joint of Case 3 (A) showing bleeding (arrow) in September 2019 while receiving albutrepenonacog alfa (rFIX-FP) with 4000 units (59.7 IU/kg) once every two weeks, and (B) disappearance of bleeding (arrow) in May 2020 while receiving rFIX-FP with 4000 units (59.7 IU/kg) once weekly.

This was a male patient in his 30s with severe hemophilia B (FIX activity <1%) and a body weight of 67 kg who works in nursing care, which involves heavy labor and night shifts. The patient had been receiving on-demand therapy with plasma-derived human coagulation FIX concentrates for many years at his former hospital, but he had either refused to start prophylactic treatment or had not been recommended prophylaxis by his former physician (the exact reason for this refusal is not clear). He visited our hospital at the end of 2015 and was introduced to prophylaxis with self-injection of 2000 units (29.9 IU/kg) of eftrenonacog alfa (a FIX product that was available in Japan from September 2014) once weekly from January 2016. However, his adherence to prophylactic self-administration of eftrenonacog alfa was not good because he was unfamiliar with the concept of prophylaxis. At a follow-up consultation in May 2018, it was discovered that he administered treatment only when he had discomfort due to bleeding, which was consequentially once every two weeks. The patient reported that he could not concentrate on his work due to bleeding in both his ankles and hip joints. Therefore, rFIX-FP (which was available in Japan from November 2016) was introduced in May 2018 on the basis that the patient would be able to have more time between injections compared with eftrenonacog alfa. The patient indicated that he wanted to receive one vial of treatment (similar to the previous treatment), so rFIX-FP was prescribed at a dose of 2000 units (29.9 IU/kg) once every two weeks. In the following month, it was confirmed that the patient had been administering his treatment, and it was suggested that his dose be increased to two vials (i.e., 4000 units or 59.7 IU/kg), which was close to the approved dose of 75 units/kg once every two weeks. The patient agreed as he was educated on the fact that prophylaxis would help protect him from bleeding, and the dose was changed. Bleeding was controlled except for a bleeding incident in the right knee in November 2018. At this time, his adherence to rFIX-FP prophylaxis had improved to almost 100%. Around October 2019, the patient reported that bleeding in the right knee frequently occurred due to an increase in night shift work. Since he understood and accepted the proposal of shortening the dosing interval of rFIX-FP to once weekly with 4000 units (59.7 IU/kg), the dosing schedule was changed. In May 2020, ultrasonography showed the disappearance of bleeding in the right knee joint (Figure 3). After the launch of vials containing 3500 units, the patient requested to switch to these due to their convenience, and he is continuing rFIX-FP prophylaxis with 3500 units (52.2 IU/kg) once a week.

## Discussion

In this case series, we introduced three patients who benefited from prophylaxis with rFIX-FP. It has been reported in recent years that arthropathy may progress as a result of repeated mild spontaneous bleeding over many years, even in people with mild (FIX activity of 5% to <40%) or moderate disease [13]. In Case 1, bleeding in the left ankle joint was improved by continued rFIX-FP prophylaxis, which allowed for the maintenance of a high trough level of FIX in a patient with moderately severe hemophilia B and advanced-stage arthropathy. Case 2 is an example of management during the administration of the antiplatelet drug to a patient with severe hemophilia B. In this patient, the dosing interval was shortened to once weekly for the management of their trough level of FIX while receiving aspirin, and antiplatelet therapy was continued safely while maintaining a trough level of 7%. In Case 3, it was difficult to continue prophylaxis with other drugs due to his adherence to treatment that required shorter intervals of administration, but switching to rFIX-FP improved the patient’s adherence to prophylactic treatment. Long-term prophylactic treatment poses a great burden on patients, particularly on patients who have intermittent or no symptoms [14,15]. However, the administration of rFIX-FP once every two weeks successfully reduced the barrier to the introduction of prophylactic treatment in this patient, who was initially not able to accept a once-weekly treatment regimen.

There are a variety of FIX products available in Japan with diverse pharmacokinetic profiles, even among the extended half-life rFIX products. The half-life of rFIX-FP is ~104 hours with single-dose administration (with an elimination half-life of 95 hours in adults) [16,17]. In comparison, eftrenonacog alfa (Alprolix®, Bioverativ Therapeutics Inc/Swedish Orphan Biovitrum) has a half-life of 78 hours (elimination half-life of 82 hours) [18,19], and nonacog beta pegol (Refixia®, Novo Nordisk) has a half-life of 115 hours [20]. These cases highlight the importance of choosing the appropriate treatment on a case-by-case basis and personalizing the proposed treatment schedule. Medical providers will need to understand the diversity of the needs and demands of patients and offer treatments that are appropriate for each individual.

## Conclusions

Our case series shows the treatment experiences after introducing prophylaxis with rFIX-FP in three Japanese individuals with moderately severe or severe hemophilia B. We highlighted the effectiveness of long-term treatment with rFIX-FP in a patient with moderately severe hemophilia B who developed arthropathy and required a treatment that maintained high trough levels of FIX to reduce bleeding. To support the safe use of aspirin, which was concomitantly administered in a patient with severe hemophilia B and an atherothrombotic disorder, regular administrations of rFIX-FP were successful in avoiding unexpected bleeding or recurrent thrombosis. Finally, in a patient who works in nursing care, which involves heavy labor and night shifts, and who had been resistant to prophylactic treatment in the past, the relatively long dosing interval of rFIX-FP allowed for improved adherence to prophylactic treatment. Taken together, these cases support the use of rFIX-FP prophylaxis in clinical practice in Japan.

## References

[1] Srivastava A, Santagostino E, Dougall A (2020). WFH guidelines for the management of hemophilia. Haemophilia.

[2] (2013). 2013 revised edition: Hemostatic treatment guidelines for hemophilia patients without inhibitors. Jpn J Thromb Haemost.

[3] Manco-Johnson MJ, Abshire TC, Shapiro AD (2007). Prophylaxis versus episodic treatment to prevent joint disease in boys with severe hemophilia. N Engl J Med.

[4] (2020). Coagulation defects in Japan Annual Report of Nationwide Survey in FY2020, the project commissioned by the Ministry of Health, Labour and Welfare. AIDS Prevention.

[5] Peters R, Harris T (2018). Advances and innovations in haemophilia treatment. Nat Rev Drug Discov.

[6] Metzner HJ, Pipe SW, Weimer T, Schulte S (2013). Extending the pharmacokinetic half-life of coagulation factors by fusion to recombinant albumin. Thromb Haemost.

[7] Santagostino E, Martinowitz U, Lissitchkov T (2016). Long-acting recombinant coagulation factor IX albumin fusion protein (rIX-FP) in hemophilia B: results of a phase 3 trial. Blood.

[8] Kenet G, Chambost H, Male C (2016). Long-acting recombinant fusion protein linking coagulation factor IX with albumin (rIX-FP) in children. Results of a phase 3 trial. Thromb Haemost.

[9] Hermans C, Marino R, Lambert C (2020). Real-world utilisation and bleed rates in patients with haemophilia B who switched to recombinant factor IX fusion protein (rIX-FP): A retrospective international analysis. Adv Ther.

[10] Oldenburg J, Yan S, Maro G, Krishnarajah G, Tiede A (2020). Assessing bleeding rates, related clinical impact and factor utilization in German hemophilia B patients treated with extended half-life rIX-FP compared to prior drug therapy. Curr Med Res Opin.

[11] Fukutake K, Taki M, Matsushita T, Sakai M, Takata A, Yamaguchi H, Karumori T (2019). Postmarketing safety and effectiveness of recombinant factor IX (nonacog alfa) in Japanese patients with haemophilia B. Haemophilia.

[12] Schutgens RE, Tuinenburg A, Roosendaal G, Guyomi SH, Mauser-Bunschoten EP (2009). Treatment of ischaemic heart disease in haemophilia patients: an institutional guideline. Haemophilia.

[13] Oldenburg J (2015). Optimal treatment strategies for hemophilia: achievements and limitations of current prophylactic regimens. Blood.

[14] Nagae C (2017). Prophylaxis therapy for hemophilia. Jpn J Thromb Haemost.

[15] Sakai M (2020). Prolonged half-life factor IX product for treatment of hemophilia B: albutrepenonacog alfa (rIX-FP: Idelvion®). Pharma Medica.

[16] (2022). European Medicines Agency: IDELVION®: Summary of product characteristics. https://www.ema.europa.eu/en/documents/product-information/idelvion-epar-product-information_en.pdf.

[17] (2022). US Food and Drug Administration: IDELVION® coagulation factor IX (recombinant), albumin fusion protein: prescribing information. https://www.fda.gov/media/96526/download.

[18] (2022). European Medicines Agency: ALPROLIX®: Summary of product characteristics. https://www.ema.europa.eu/en/documents/product-information/alprolix-epar-product-information_en.pdf.

[19] (2022). US Food and Drug Administration: ALPROLIX® coagulation factor IX (recombinant), Fc fusion protein: Prescribing information (2014). https://www.fda.gov/media/88119/download.

[20] (2022). European Medicines Agency: Refixia: Summary of product characteristics. https://www.ema.europa.eu/en/documents/product-information/refixia-epar-product-information_en.pdf.

